# Antiretroviral Therapy Outcomes among Adolescents and Youth in Rural Zimbabwe

**DOI:** 10.1371/journal.pone.0052856

**Published:** 2012-12-20

**Authors:** Helen Bygrave, Judith Mtangirwa, Kwenzakwenkosi Ncube, Nathan Ford, Katharina Kranzer, Dhodho Munyaradzi

**Affiliations:** 1 Southern Africa Medical Unit, Médecins Sans Frontières, Cape Town, South Africa; 2 Ministry of Health and Child Welfare Zimbabwe, Buhera District, Zimbabwe; 3 Centre for Infectious Disease Epidemiology and Research, University of Cape Town, Cape Town, South Africa; 4 Clinical Research Unit, London School of Hygiene and Tropical Medicine, London, United Kingdom; 5 Medical Unit, Médecins Sans Frontières, Harare, Zimbabwe; Indiana University and Moi University, United States of America

## Abstract

Around 2 million adolescents and 3 million youth are estimated to be living with HIV worldwide. Antiretroviral outcomes for this group appear to be worse compared to adults. We report antiretroviral therapy outcomes from a rural setting in Zimbabwe among patients aged 10–30 years who were initiated on ART between 2005 and 2008. The cohort was stratified into four age groups: 10–15 (young adolescents) 15.1–19 years (adolescents), 19.1–24 years (young adults) and 24.1–29.9 years (older adults). Survival analysis was used to estimate rates of deaths and loss to follow-up stratified by age group. Endpoints were time from ART initiation to death or loss to follow-up. Follow-up of patients on continuous therapy was censored at date of transfer, or study end (31 December 2008). Sex-adjusted Cox proportional hazards models were used to estimate hazard ratios for different age groups. 898 patients were included in the analysis; median duration on ART was 468 days. The risk of death were highest in adults compared to young adolescents (aHR 2.25, 95%CI 1.17–4.35). Young adults and adolescents had a 2–3 times higher risk of loss to follow-up compared to young adolescents. When estimating the risk of attrition combining loss to follow-up and death, young adults had the highest risk (aHR 2.70, 95%CI 1.62–4.52). This study highlights the need for adapted adherence support and service delivery models for both adolescents and young adults.

## Background

Around 2 million adolescents and 3 million youth are estimated to be living with HIV worldwide. Of these, 65% are young girls, and the majority are living in sub-Saharan Africa. 41% of all new infections in 2009 occurred in the 15–24 age group [1]. Recognising the growing need UNICEF recently released a report highlighting the need for HIV prevention and treatment programmes to focus specific efforts towards young people living with HIV/AIDS [2]. Data from both Western settings [3] and studies from resource-limited settings [4], [5] suggest that antiretroviral treatment outcomes for young people are worse compared to adults.

Developing a clear understanding of the outcomes, adherence and behavioural needs of this group is essential [6]–[7]. Programmes have been developed in Western settings to provide targeted support for adolescents and youth [8], but it is unclear how applicable these models are in resource-limited settings.

In this study, we compare retention in care in young people receiving antiretroviral therapy in a rural setting in Zimbabwe in order to determine which age groups are at greatest risk of adverse outcomes in this setting.

## Methods

This study was carried out in the rural district of Buhera (population 220,000), Manicaland, where the medical aid agency Médecins Sans Frontières (MSF) has supported the Zimbabwean Ministry of Health and Social Welfare since the end of 2004. The program provides ART to over 16,000 patients via 25 decentralised primary health care clinics. HIV prevalence in 2009 in the 15–24 age group was 7.7% in females and 2.9% in males. Initiation of ART during the period analysed was performed by doctors with ongoing follow up performed by nurses according to national guidelines during the study period. The standard first line therapy used during the period analysed was stavudine, lamivudine, and nevirapine. Eligibility criteria during the period analysed were those patients with a CD4<200 cells/mm^3^, stage 4 conditions or those with TB. Three sessions of preparatory counselling were given prior to ART initiation followed by sessions at weeks two, four, twelve and then three monthly during the first year on ART. All of the decentralised primary health care sites provided HIV and ART care to all age groups and no specialised paediatric or adolescent services were available during the period analysed.

The analysis was performed on data collected as part of routine programme monitoring and evaluation. Clinicians entered clinical information into structured clinic visit forms at each patient assessment and data were then prospectively entered into an electronic patient register by dedicated data enterers. All data was anonymized using a unique patient registration number. The dataset included all patients aged between 10 and 30 years at the time of ART initiation who started ART between 1^st^ of January 2005 and 30^th^ of June 2008 according to the previously stated eligibility criteria. The cohort was stratified into four age groups in line with international definitions [1]: 10–15 (young adolescents) 15.1–19 years (adolescents), 19.1–24 years (young adults) and 24.1–29.9 years (older adults). Loss to follow-up was defined as non-attendance for a period of three months after the last ART prescription had been completed. Death was defined as any death while taking treatment. Both mortality and loss to follow up were confirmed through family reporting and tracing performed by support groups and a community-based NGO.

Baseline characteristics were described using median and interquartile ranges (IQRs) for continuous variables and counts and percentages for categorical variables. Survival analysis was used to estimate rates of deaths and loss to follow-up stratified by age group. Entry time into the cohort was the date of ART initiation. Endpoints were the time from ART initiation to death or loss to follow-up. Follow-up of patients on continuous therapy was censored at date of transfer, or study end (31 December 2008). Sex-adjusted Cox proportional hazards models were used to estimate hazard ratios for different age groups. Hazard proportionality was assessed by analysis of scaled Schoenfeld residuals. Sensitivity analyses were performed to adjust for baseline immune deficiency. Hazard ratios comparing different age groups were estimated adjusted for baseline CD4 count (<100, 101–200, >200) in two ways: (i) using the complete data set and (ii) creating a separate category for missing CD4 counts. All analyses were conducted in STATA version 11.

This study used routine programme data and analysis of this data received approval from the Independent Ethics Review Board of Médicins Sans Frontières.

## Results

A total of 898 patients were included in the analysis. Table 1 shows the baseline characteristics of the cohort by age group. There was an equal proportion of males and females among the young adolescents (49.3% male) and adolescents (47.1% male) suggesting a higher proportion of perinatally infected children in this group as in adult services a higher proportion of women tend to access care; in the young adults and adult age groups the majority of patients (>80%) were female. Median duration on ART was 441 days (IQR 257–643), and this was highest for young adolescents (484 days, IQR 334–755). CD4 cell counts were missing for 294 (32.7%) patients. The proportion of missing CD4 cell counts was similar across all age categories. Median CD4 cell count was <200 cells/mm^3^ across all age categories, and lowest for adolescents (median CD4 102 cells/mm^3^, IQR 21–184).

**Table 1:** Baseline Characteristics at ART Initiation.

	Young adolescents	Adolescents	Young adults	Adults	Total
	(10–15 years)	(15.1–19 years)	(19.1–24 years)	(24.1–29.9 years)	
	(n = 221)	(n = 85)	(n = 143)	(n = 449)	(n = 898)
Sex, n (%)					
Female	112 (50.7)	45 (52.9)	123 (86.0)	361 (80.4)	641 (71.4)
Male	109 (49.3)	40 (47.1)	20 (14.0)	88 (19.6)	257 (28.6)
Age, median (IQR)	12.3 (11.2–13.6)	16.4 (15.6–17.7)	22.4 (21.0–23.5)	27.1 (25.9–28.2)	
CD4 counts					
Missing, n (%)	68 (30.8)	29 (34.1)	53 (37.1)	144 (32.1)	294 (32.7)
Available, n (%)	153 (69.2)	56 (65.9)	90 (62.9)	305 (67.9)	604 (67.3)
Medan (IQR)	154 (50–265)	102 (21–184)	111 (41–230)	111(39–224)	119 (44–236)
<100 cells/uL, n (%)	54 (35.3)	27 (48.2)	44 (48.9)	140 (45.9)	265 (43.9)
100–200 cells/uL, n (%)	36 (23.5)	18 (32.1)	14 (15.6)	65 (21.3)	133 (22.0)
>200 cells/uL, n (%)	63 (41.2)	11 (19.6)	32 (35.6)	100 (32.8)	206 (34.1)
Year of Initiation				40 (8.9)	70 (7.8)
2004–2005 (%)	20 (9.1)	5 (5.9)	5 (3.5)	73 (16.3)	156 (17.4)
2006 (%)	44 (19.9)	15 (17.7)	24 (16.8)	217 (48.3)	451 (50.2)
2007 (%)	110 (49.8)	44 (51.8)	80 (55.9)	119 (26.5)	221 (24.6)
2008 (%)	47 (21.3)	21 (24.7)	34 (23.8)		
Time on ART (days), median (IQR)	484 (334–755)	434 (259–665)	405 (216–559)	441 (240–625)	441 (257–643)

**Figure 1:**
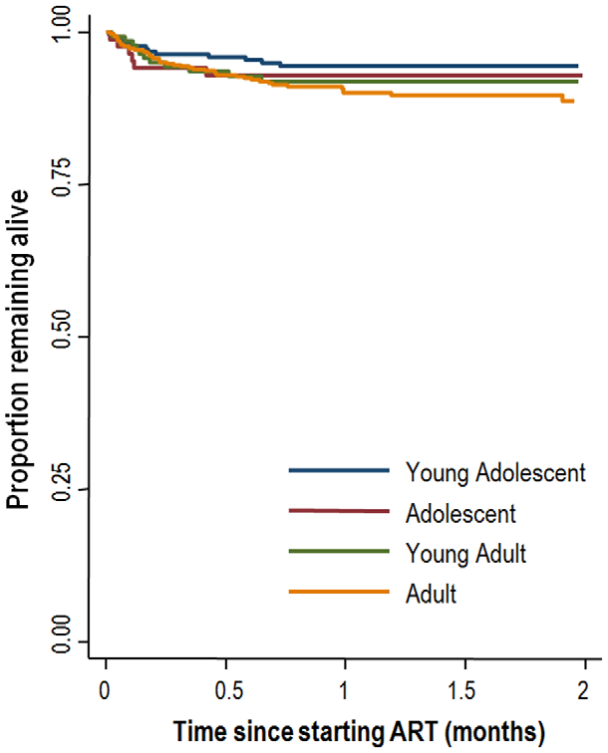
Kaplan-Meier plots of deaths and loss to follow-up stratified by age category.

**Figure 2:**
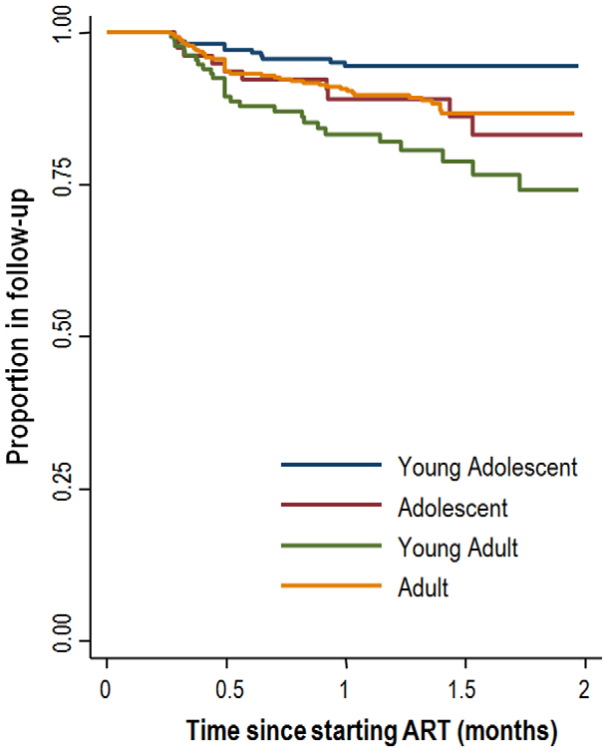
Kaplan-Meier plots of loss to follow-up stratified by age category.

Overall, the rate of death was 6.2 (95%CI 4.9–7.7) per 100 person years (table 2) with the highest rates in the first months of treatment (Figures 1 & 2). Adults had the highest rate of death at 7.7 (95%CI 5.8–10.4) per 100 person years, while young adolescents had the lowest death rate at 3.6 (2.0–6.3) per 100 person years. In contrast, loss to follow-up was highest among the young adults, at 16.8 (95%CI 11.6–24.3) per 100 person years, and lowest in the young adolescent age group, at 4.2 (2.5–7.0) per 100 person years. Adjusted hazard ratios for death were highest in adults (2.25, 95%CI 1.17–4.35) compared to young adolescents (table 3), with no significant differences observed in the hazard of death comparing young adolescents and adolescents or young adults. Results were similar when adjusting for baseline immune deficiency using a separate category for missing CD4 cell count or restricting the analysis to the complete dataset. In contrast, young adults and adolescents had a 2–3 times higher risk of loss to follow-up than young adolescents (aHR 3.35, 95%CI 1.73-6.49 for young adults and aHR 2.54, 95%CI 1.17–5.49 for adolescents). When estimating the risk for either loss to follow-up or death young adults had the highest risk (aHR 2.70, 95%CI 1.62–4.52). The results remained largely unchanged when adjusting for baseline CD4 count both when a separate category was used for missing CD4 counts or when the analysis was restricted to the complete dataset.

**Table 2:** Rates of death and Loss to follow-up.

	Death			Loss to follow-up		
	Events	Person-years	Rate (95% CI) per 100 person-years	Events	Person time (years)	Rate (95% CI) per 100 person-years
Young adolescents	12	336	3.6 (2.0–6.3)	14	336	4.2 (2.5–7.0)
Adolescents	6	111	5.4 (2.4–12.1)	12	111	10.9 (6.2–19.1)
Young adults	11	166	6.6 (3.7–11.9)	28	166	16.8 (11.6–24.3)
Adults	45	582	7.7 (5.8–10.4)	45	582	7.7 (5.8–10.4)
Total	74	1200	6.2 (4.9–7.7)	99	1200	8.3 (6.8–10.1)

**Table 3:** Hazard ratios for death, loss to follow-up and the death and loss to follow-up combined comparing young adolescents with adolescents, young adults and adults.

	Hazard ratio for death adjusted for (95% CI)			Hazard ratio for loss to follow-up adjusted for (95%CI)			Hazard ratio for death and loss to follow-up adjusted for (95%CI)		
	Gender	Gender and CD4 count using the complete dataset	Gender and CD4 count coding missing data as a separate category	Gender	Gender and CD4 count using the complete dataset	Gender and CD4 count coding missing data as a separate category	Gender	Gender and CD4 count using the complete dataset	Gender and CD4 count coding missing data as a separate category
Young adolescents	1	1	1	1	1	1	1	1	1
Adolescents	1.40 (0.53–3.74)	1.34 (0.45–4.03)	1.22 (0.46–3.26)	2.54 (1.17–5.49)	2.55 (1.05–6.20)	2.34 (1.08–5.08)	2.00 (1.10–3.65)	1.95 (0.98–3.87)	1.81 (0.99–3.32)
Young adults	1.82 (0.78–4.24)	1.85 (0.88–4.05)	1.58 (0.68–3.66)	3.35 (1.73–6.49)	2.34 (1.04–5.28)	3.13 (1.61–6.08)	2.70 (1.62–4.52)	2.13 (1.15–3.96)	2.46 (1.47–4.10)
Adults	2.25 (1.17–4.35)	1.88 (0.94–2.99)	1.98 (1.02–3.81)	1.62 (0.88–2.99)	1.46 (0.72–2.95)	1.52 (0.82–2.81)	1.88 (1.20–2.94)	1.65 (0.98–2.77)	1.72 (1.10–2.69)

## Discussion

There is growing recognition that adolescents have greater challenges to adherence on ART compared to adults. However, published studies from cohorts of this age group from resource-limited settings are limited and little consistency in the age categories used in published studies, making comparisons difficult [4], [9]. In addition the largely unrecognised epidemic of late progressors in the young and older adolescent age groups is evidenced by the almost equal sex distribution in these age groups in our setting. Across Southern Africa an increasing pool of survivors from mother-to-child transmission programmes are entering into adolescence, with data suggesting that up to one third of HIV infected infants, likely to have been infected during the breast feeding period, are “slow progressors”” with a median survival of greater than ten years. In Zimbabwe, mathematical modelling estimates that deaths among untreated slow progressors will increase from 8000 per year in 2008 to a peak of 9700 per year in 2014. Hence, identifying these children and addressing their needs once on treatment is an essential challenge for antiretroviral therapy (ART) programmes in the coming years [3].

In this study, from a rural primary health care programmes in a resource-limited settings, the age group with the worst outcomes were the young adults (19.1–24 years). Cited challenges to medication adherence in this group include side effects, appointment schedules that interfere with daily schedules, depression, stigmatisation, fears about disease transmission, transfer to adult HIV services [10], and disclosure of HIV status [11], [12]. In this setting partial disclosure is aimed for between the ages of seven to nine with full disclosure completed no later than age twelve. While no formal qualitative surveys have been done in this programme, feedback from experienced counsellors working in these sites suggested that context specific challenges to retention in care for the young adult group also highlighted during informal focus included: the transition out of education, the need to move away in order to seek work (moving to an urban setting or moving across a border to South Africa or Botswana); and entering into more serious relationships and marriage where new disclosure was needed.

Other factors that may have impacted on rates of retention rates during the period analysed included the fact that decentralisation to the network of primary care clinics was gradually being implemented with patients having to travel up to 3 hours to reach a clinic. In addition, the economic and political instability experienced in Zimbabwe during this period may have led to patients defaulting due to inability to pay for transport or due to forced movement out of the district.

In a survey carried out between 1998 and 2005 in Manicaland, Zimbabwe (the same province as this study site) the median age at first sex and first marriage was 18.5 years and 21.4 years for men and 18.2 years and 18.5 years for women respectively [13]. Marriage and giving birth, particularly to a first born, may be a reason for patients to move away from their original clinic. Many women will travel to be near a parent or in laws depending on cultural norms and hence be lost within the ART cohort.

Various counselling and service delivery models of care have been developed in western settings targeting adolescents. These have also included the development of “transitioning protocols” from specialised adolescent clinics to the standard adult services [8]. However, in resource-limited settings where counsellors are often lay people with relatively low levels of education, it may not be feasible to implement specialised counselling techniques.

Simple steps to addressing the major challenges to retention in care should be incorporated into existing counselling tools and service delivery models. One example being employed in one South African adolescent project is incorporating the theme of “life steps” into routine counselling that aims to address the major challenges around having to seek work and entering into new relationships. Whether peer support for this age group is helpful is questionable as when asked, many stated that they would prefer to be counselled by someone older than themselves.

Adapting both provision of ART and monitoring and evaluation tools to the challenge of mobility of this age group should also be integrated into programme implementation. Counsellors and nurses should proactively and routinely ask about travel plans for work, education or social reasons and allow for provision of longer drug supplies and a degree of flexibility in often rigid follow up schedules. Being proactive in this way should allow formal transfers out to be organised to alternative ART sites when needed. In addition it is essential to provide a clearly documented HIV history to the patient, the so called patient passport, enabling the health professional at the new site to continue care in an appropriate manner.

In addition to adapting approaches to the socioeconomic challenges in this age group, simplification of drug delivery may also be another route to aid adherence. Already the introduction of guidance to use a once-daily, fixed-dose combination of tenofovir combined with lamivudine and efavirenz from the age of twelve is expected to simplify adherence and reduce side-effects [13]. In countries where such optimized regimens are still unavailable for the general population due to cost concerns it may be reasonable to consider prioritizing their use in this high risk age group. Long acting formulations that are in development could also be ideal to pilot for these age groups. The use of mobile health technologies and pre-existing social network platforms may be another attractive means of giving additional support mechanisms to this vulnerable age group.

As with any observational study there were several limitations to the analysis. Incomplete baseline CD4 data did not allow for appropriate adjustment. Adjustment for baseline CD4 count using a separate category for missing values might have introduced bias. This is particularly true in situations where the percentage of missing data is high [14]–[17]. Restricting the analysis to records with complete CD4 count data will also result in bias except when the variable is missing completely at random. The proportion of missing CD4 counts was similar across age groups and outcomes (death and loss to follow-up). A high proportion (32.8%) of CD4 counts were missing for operational reasons, as the country was going through a period of civil unrest with breakdown of infrastructure. Therefore CD4 count data were likely to be missing completely at random and analysis of the complete case scenario can be expected to result in unbiased results. Incomplete outcome verification for patients lost to follow up may lead to possible misclassification of true death as loss to follow up [18]. Nevertheless, when deaths and losses were combined as a single adverse outcome (attrition), young adults still did worse. Finally, the fact that a proportion of adolescents may have been perinatally infected may reflect a survival bias in this group.

In conclusion, this analysis highlights the young adult group as being at highest risk of being lost from care compared to adolescent and adults in a resource poor rural setting. Adapting adherence support and service delivery models for this group should be a priority to avoid treatment interruptions, development of resistance and increased morbidity within this group.
